# Large language models and brain-inspired general intelligence

**DOI:** 10.1093/nsr/nwad267

**Published:** 2023-11-03

**Authors:** Bo Xu, Mu-ming Poo

**Affiliations:** Institute of Automation, Chinese Academy of Sciences, China; CAS Center for Excellence for Brain Science and Intelligence Technology, China

The emergence of large language models (LLMs) such as ChatGPT/GPT-4 and their stunning performance in generative tasks heralds the beginning of a new era of artificial general intelligence (AGI). The LLMs have shown amazing generalization ability in natural language processing, computer vision and other generative tasks [1]. These models point to a feasible approach for developing AGI. To a large extent, they have passed the classical Turing test and their capability is still growing without an upper bound identified. Further development of LLMs could contribute to large embodiment models that have more general and powerful capabilities through collaboration with physical robotics. These embodiment models will be capable of simulating human intelligence, possessing not only the capacity for language generation and comprehension, but also the ability to perceive and learn through interactions with their environments [2]. A variety of scientific, educational and commercial applications based on LLMs are emerging and they have sparked limitless imagination about the future AI environment for humans. In the meantime, there are serious concerns about their potential negative impacts on many aspects of the human society and the future of humanity.

One of the biggest concerns is that current LLMs consume a huge amount of energy. By constructing complex network structures with super-large numbers (10^11^) of adjustable parameters and fed with a huge amount of data (10^12^ tokens—one word can be encoded into one or several tokens [3]), training of LLMs consumes astonishing amounts of energy. Furthermore, compared with the one-time consumption in the training process, the inference process can be repeated over and over again by different users and thus represents iterative consumption that uses much more energy. In the case of ChatGPT, for example, it has been calculated that its cost is ∼1.7 million US dollars when using cloud-based servers for training. By contrast, in a single inference by a user, it costs ∼0.177 cents to process the information of 1000 tokens [4]. However, it will take >4 million Nivida A100 GPU if only Google search were replaced by the ChatGPT service, based on a report from SemiAnalysis [5], let alone more LLM services that will cause a catastrophic increase in the inference cost. The huge cost in the training and inference phases of LLMs is caused by data-driven computational adjustments and parallel inferences of a large number of connectivity parameters. This energy cost undoubtedly constrains the commercialization of LLMs and their service to the infrastructure of various sectors of society.

A promising approach to solving the energy problem is brain-inspired machine learning and computing. Compared with LLMs, the human brain performs much more complex tasks, comprises a larger network (with 86 billion neurons and trillions of synapses), but only consumes ∼20 W of power. The highly efficient neural network in the human brain is endowed with the evolutionary process, which provides the diversity of cell types and overall connectivity patterns, and is further refined during postnatal brain development, when the most efficient connectivity patterns are formed based on the individual's interaction with the environment. Even if we are unable to produce a ‘child machine’ as Turing once proposed [6], AGI could learn from the mature brain in two aspects—the neural network architecture and computational algorithms.

The artificial neural networks in the past had been inspired somewhat by brain networks, such as the layered and modular structure and modifiable weights of synaptic connections. Yet brain networks achieve their efficiency by adding more diverse neuronal types and selective connectivity among various types of neurons in distinct modules within the network, rather than simply adding more neurons, layers and connections, such as that underlying deep-learning networks used in LLMs. Brain network connectivity from local network motifs to global topologies has already inspired convolutional and recurrent operators, and more powerful neural operators such as various revised transformer operators. Studies of the whole-brain spatial transcriptome (for identifying diverse neuronal subtypes) and mesoscopic connectome (for defining cell type-specific connectivity) [7] are now providing a rich database for further design of more efficient network architectures of LLMs.

Synaptic connections are known to be locally modulated by neural activity via many forms of synaptic plasticity, such as short-term and long-term synaptic potentiation and depression. Neuromodulators in the brain could act globally at many synapses to modify the capacity and property of local activity-dependent synaptic plasticity. Computationally costly backpropagation algorithms, the core method for many existing AI networks, could be improved or even replaced by biologically plausible learning algorithms. The learning algorithms could incorporate diverse forms of activity-dependent synaptic plasticity found in the brain at both local and global levels, such as spike timing-dependent plasticity, self-organized propagation of plasticity, neuromodulator-dependent metaplasticity and short-term vs. long-term memory storage rules that set the stability of synaptic weight changes within the network [8,9]. The conventional gradient descent approaches merged with the above plasticity-based learning rules could embrace the core principle of backpropagation while overcoming many of its problematic implementation requirements, such as feedback alignment, target propagation and those approximations to a gradient descent called the NGRAD hypothesis [10]. Development of new computational algorithms for connectivity adjustment could make LLMs more efficient and flexible, and it is a challenging task that requires in-depth interaction between neuroscience and AI.

Developing new network architectures and learning algorithms together will enable new forms of brain-inspired computing (BIC), i.e. implementing bio-inspired spiking neural networks (SNNs) on neuromorphic chips [11–13]. At the network and algorithmic levels, SNNs use biologically plausible spiking neurons with rich spiking patterns as the basic computational units and can more easily exploit multiscale synaptic plasticity. At the hardware level, neuromorphic chips require a new generation of non-von Neumann computing architecture by drawing on the event-driven sparse computing, highly parallel operation, as well as co-localized processing and memory of the brain to reduce the hardware power cost [14]. A fascinating nature of BIC is that only a small fraction of spiking neurons are activated to participate in sparse addition operations when inference is performed. Such sparse computing is well suited for reducing the training and inference costs of large AI models.

While developing green and sustainable low-power AI is a pressing issue for large AI models at present, there are growing concerns that future development of AGI could greatly impact human society in a manner that could be detrimental. Concentration of the power of designing and using LLMs to a few large corporations, which are mainly driven by profits, and to individuals whose motives are uncertain could lead to scenarios no less dangerous than the unregulated use of nuclear power or biotechnology. This calls for an effective global governance of the future development of AGI, with a long-term view and consensus among all governments and industries that guide AGI development in the coming decades. This is not going to be easy, given the existence of political, social and cultural differences, but it is a step that must be taken for the survival of humanity.
